# Correction: Quantification of gait parameters in freely walking wild type and sensory deprived *Drosophila melanogaster*

**DOI:** 10.7554/eLife.00565

**Published:** 2013-02-11

**Keywords:** walking behavior, coordination, proprioception, sensory feedback, gait analysis, motor neuron, D. melanogaster

César S Mendes, Imre Bartos, Turgay Akay, Szabolcs Márka, Richard S Mann

Mendes CS, Bartos I, Akay T, Márka S, Mann RS. 2013. Quantification of gait parameters in freely walking wild type and sensory deprived *Drosophila melanogaster*. *eLife* **2**:e00231. doi: http://dx.doi.org/10.7554/eLife.00231. Published 8 January 2013

In the first sentence of the abstract, ‘multi-legged in vertebrates’ has been corrected to ‘multi-legged invertebrates’.

In the vertical axis of Figure 7, panel B, ‘Step lenght’ has been corrected to ‘Step length’.

The article has been corrected accordingly.

